# A bibliometric analysis of transcranial alternating current stimulation

**DOI:** 10.3389/fnins.2024.1409492

**Published:** 2024-08-02

**Authors:** Cheng-Fan Wu, Chao Shen, Zhao-Di Wang, Yan Gong, Lu-Han Zhou, Wen-Jun Qian, Tong Tang

**Affiliations:** ^1^Department of Rehabilitation Medicine, The Affiliated Suzhou Hospital of Nanjing Medical University, Suzhou, China; ^2^The Fourth Rehabilitation Hospital of Shanghai, Shanghai, China

**Keywords:** bibliometrics, transcranial alternating current stimulation, CiteSpace, oscillations, visual analysis

## Abstract

**Background:**

Transcranial alternating current stimulation (tACS) can apply currents of varying intensity to the scalp, modulating cortical excitability and brain activity. tACS is a relatively new neuromodulation intervention that is now widely used in clinical practice. Many papers related to tACS have been published in various journals. However, there are no articles that objectively and directly introduce the development trend and research hotspots of tACS. Therefore, the aim of this study is to use CiteSpace to visually analyze the recent tACS-related publications, systematically and in detail summarize the current research hotspots and trends in this field, and provide valuable information for future tACS-related research.

**Material and methods:**

The database Web of Science Core Collection Science Citation Index Expanded was used and searched from build to 4 August 2023. Using the CiteSpace to analyze the authors, institutions, countries, keywords, co-cited authors, journals, and references.

**Results:**

A total of 677 papers were obtained. From 2008 to 2023, the number of publications shows an increasing trend, albeit with some fluctuations. The most productive country in this field was Germany. The institution with the highest number of publications is Carl von Ossietzky University of Oldenburg (*n* = 50). According to Bradford’s law, 7 journals are considered core journals in the field. Herrmann, CS was the author with the most publications (*n* = 40), while Antal, A was the author with the highest number of co-citations (*n* = 391) and betweenness centrality (*n* = 0.16). Disease, neural mechanisms of the brain and electric stimulation are the major research areas in the field. The effect of tACS in different diseases, multi-site stimulation, combined treatment and evaluation are the future research hotspots and trends.

**Conclusion:**

tACS has research value and research potential, and more and more researchers are paying attention to it. The findings of this bibliometric study provide the current status and trends in the clinical research of tACS and may help researchers to identify hotspots s and explore new research directions in this field.

## Introduction

Transcranial alternating current stimulation (tACS) is a widely used method of non-invasive brain stimulation (NIBS) (Rampersad et al., 2019). This technology delivers electrical currents to the brain through the scalp, mimicking the naturally occurring rhythms of the brain’s electrophysiological activity and generating endogenous oscillations (Ruffini et al., 2013; Wischnewski et al., 2023). Oscillations can modulate connections and communications between the cerebral cortex, affecting the balance of synchronized and periodic fluctuations between excitatory and inhibitory circuits of neuronal populations, resulting in therapeutic effects (Bonnefond et al., 2017; Preisig et al., 2021; van Bree et al., 2021). At present, tACS has being used extensively in the study of various diseases.

TACS is commonly used in Alzheimer’s disease (AD) (Benussi et al., 2021; Altomare et al., 2023; Benelli et al., 2023; Nissim et al., 2023), depression (Biacková et al., 2023; McAleer et al., 2023a; Pan et al., 2023; Raymond et al., 2023), chronic insomnia (Wang et al., 2020b; Shan et al., 2023), mental illness and so on (Ahn et al., 2019; Raymond et al., 2023). One study found that tACS was effective in improving negative symptoms in neurodivergent patients (Chang et al., 2021). However, the results were inconsistent in terms of improving cognitive outcomes (Mellin et al., 2018). In studies of patients with Parkinson’s disease (PD), tACS was found to improve motor cortex plasticity and enhance GABA-Aergic transmission (Guerra et al., 2023). In a meta-analysis of healthy individuals, it was found that tACS stimulation increased cortical excitability when the stimulation intensity exceeded 1 mA, but the optimal stimulation parameters require further study (Wischnewski et al., 2019). Currently, there are many articles about tACS, but the efficacy and neural mechanisms of tACS in various diseases are not fully understood. Understanding these issues is crucial for realizing the precise treatment of tACS. To gain a comprehensive understanding of the research trends in the field of tACS and to predict future development frontiers, this study conducted a bibliometric analysis of tACS-related studies.

Bibliometric analysis is a plausible method for the qualitative and quantitative evaluation of the publications on a specific topic (Nakagawa et al., 2019). Bibliometric analysis transforms abstract data into intuitive graphics using computer image processing techniques. Through visualized map, research contributions are presented objectively according to countries, institutions, journals, authors, references and keywords in a given research field, and reveal the hotspots and trends in a specific research field (Zhao and Li, 2023). In other words, bibliometrics can rigorously interpret unstructured data on specific medical research topics. At present, there are bibliometric analyses of tDCS (Sun et al., 2022; Zhou et al., 2023), transcranial magnetic stimulation (TMS) (Lawson McLean, 2019), and neuromodulation interventions (Sabé et al., 2023), but the bibliometric analysis of tACS has not been found.

Based on the Web of Science Core Collection (WoSCC) database, this research adopts CiteSpace knowledge mapping software to understand and compare the basic situation, research hotspots and development trends of tACS from the perspective of visualization. Its findings aim to offer guidance and novel insights for related research.

We used CiteSpace software to study tACS-related research spots from 2008 to 2023. This study provides an objective description of the scientific field in the following three areas.

(1) From 2008 to 2023, quantify the amount of tACS-related annual research output in WoSCC.

(2) CiteSpace software was used to identify the principal authors, journals, countries, references, and institutions that published tACS research illustrate the knowledge base of tACS.

(3) Through keyword co-occurrence, keyword clustering and keyword burst analysis, the knowledge framework and hot spot evolution of tACS were explored, and the research on emerging topics was promoted. In summary, these three objectives cover the current state of research and recent trends in tACS.

## Materials and methods

### Data source and search strategy

Data were obtained from the WoSCC (Deng et al., 2022; Bai et al., 2023), which contains multidisciplinary, high-impact, international, and comprehensive academic journals and is a suitable database for bibliometric analysis. Considering that the database is updated daily, all the data were retrieved and exported from WoSCC on 4 August 2023 to avoid the bias. The search strategy was: [TS = (“transcranial alternating current stimulation” OR “tACS”)]. A preliminary search yielded 2,142 articles.

### Inclusion and exclusion criteria

**FIGURE 1:**
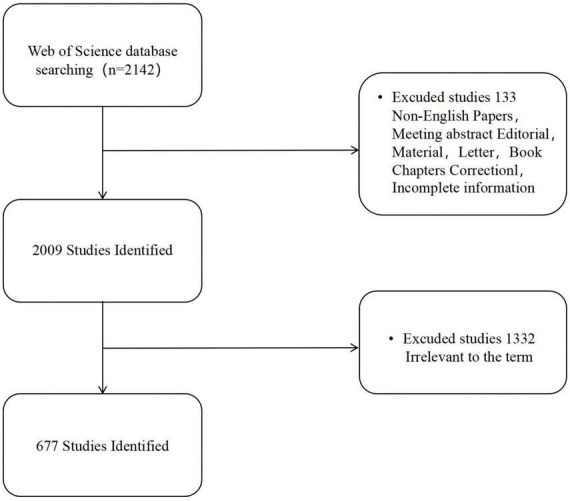
Database search flow chart (Software: WPS Office 2023, Kingsoft, China).

Only English-language literature published as “article” and “review” was included. Exclusion criteria were editorial materials, letters, conference abstracts, news items, book chapters, corrections, proceedings papers or early access and other types of literature. And repeated publications, literatures with incomplete indicators, literatures unrelated to the tACS research topic are also excluded. The retrieved literature was imported into EndNote X20 literature management software. 2 researchers independently read the titles and abstracts of the literature, excluded irrelevant literature and cross-checked. If there was a difference of opinion, the third researcher assisted in the decision. Finally, 677 documents were included in the final screening (Figure 1).

### Data extraction

Select the 677 eligible references as “fully recorded and cited references.” Export them to “plain text file format,” and rename them to “download_. Txt” to ensure that the CiteSpace (6.2.R4) software can read the records correctly.

### Data analysis

Microsoft Office Excel 2021, CiteSpace (6.2.R4) software was used for the bibliometric analysis. The graphs generated by Microsoft Office Excel show the number of articles published in different years. Using the CiteSpace to analyze the authors, institutions, countries, keywords, co-cited authors, journals, and references. CiteSpace is a Java-based application that visualizes the structure, patterns, and distribution of the literature through visualization (Chen, 2004, 2017). This study uses CiteSpace6.2.R4 (64–bit), which uses nodes and links to generate visualization knowledge maps. Each node in the map represents an element to be analyzed and the size of the node represents the frequency of citations (Chen et al., 2023). The color of the colored rings and lines around a node represents the year in which the object or relationship first appeared in the literature, with different colored nodes representing different years (Song et al., 2023). The connecting lines between nodes represent co-occurrence or co-citation relationships between two objects (Ye et al., 2023), and the thickness of the lines represents the strength of the relationship between the objects. Purple circles around certain nodes represent the betweenness centrality (BC), which is a measure of the importance of nodes in a network. BC ≥ 0.1 is usually considered to be a turning point in a domain (Sun et al., 2022).

In mapping the visualization knowledge maps, we followed the main procedural steps of CiteSpace, including time slicing, thresholding, modeling, pruning, merging and mapping. The parameters are set as follows: Time Slicing (2008–2023); Year per Slice (1); Term Source (Title/Abstract/Author Keywords/Keywords Plus); Node Type (Author/Institution/Country/Keyword/Reference/Cited Author/Cited Journal); Top N (50); Pruning (Pathfinder/Pruning sliced networks); Visualization (Cluster View-Static/Show Merged Network).

## Results

### Annual quantitative distribution of publications

**FIGURE 2:**
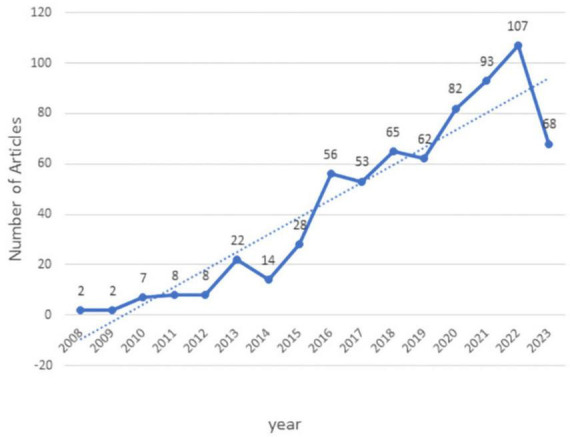
Annual number of publications about tACS. Microsoft Excel 2021 software was used for graphing the annual circulation of publications (Software: Microsoft Excel 2021, Microsoft, USA).

The number of annual publications is shown in Figure 2. Between 2008 and 2023, a total of 677 articles have been published in this field. Despite small fluctuations in 2014, 2017, and 2019, there was an overall increase in the number of tACS articles. The publication growth trend can be divided into two phases: the initial phase (2008–2012). Although the concept of “tACS” attracted scientific attention during this period, there were few publications and the number of publications grew slowly. Rapid growth phase (2014–2023). In this phase, publication output generally increases from year to year, with the exception of a few years of decline (Figure 2).

### Countries

**FIGURE 3:**
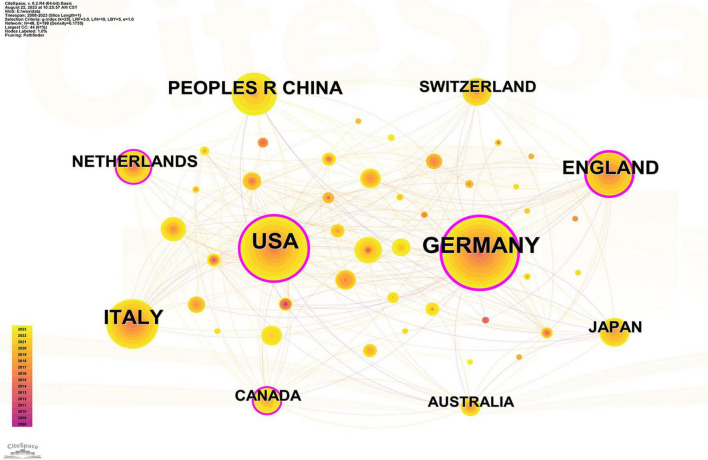
Country collaborative network analysis. Time slicing: January 1999 to December 2023, Slice length: 1 year, Node types: Country, g-index (*K* = 25), Pruning: Pathfinder, *N* = 4, *E* = 145 (Software: CiteSpace 6.2.R4, Drexel University, Philadelphia, USA).

As shown in Table 1, a total of 48 countries published studies in the included articles. Among them, Germany (205) published the highest number of articles on tACS, followed by the United States (USA) (168), Italy (108), the United Kingdom (UK) (88), and China (76) (Table 1). The top 3 countries were Germany (BC = 0.4), the UK (BC = 0.33) and the USA (BC = 0.21) in terms of BC. Furthermore, while Canada (BC = 0.18) and the Netherlands (BC = 0.15) did not make it into the top 5 countries with the most published articles, they ranked high in terms of BC value (Figure 3).

**TABLE 1:** Top 10 countries by publications.

Rank	Country	Count	BC	Year
1	Germany	205	0.40	2008
2	USA	168	0.21	2009
3	Italy	108	0.10	2011
4	England	88	0.33	2008
5	China	76	0.05	2009
6	Netherlands	44	0.15	2010
7	Japan	38	0.03	2009
8	Switzerland	38	0.03	2012
9	Canada	33	0.18	2012
10	Australia	28	0.01	2009

BC, betweenness centrality.

### Institutions

**FIGURE 4:**
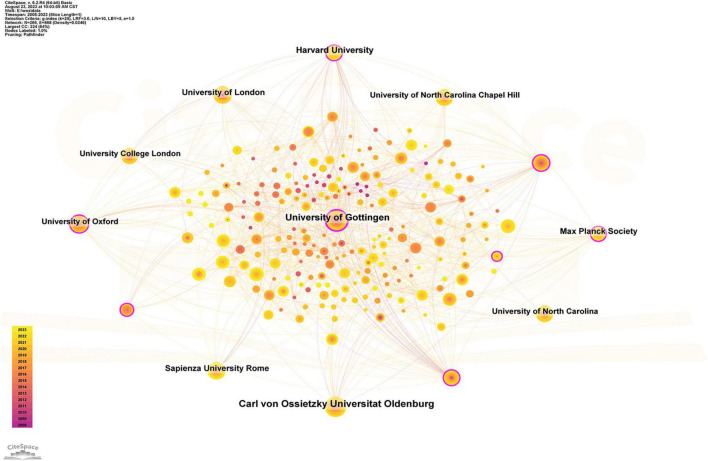
Institution collaborative network analysis. Time slicing: January 1999 to December 2023, Slice length: 1 year, Node types: Institution, g-index (*K* = 20), Pruning: Pathfinder, *N* = 293, *E* = 727 (Software: CiteSpace 6.2.R4, Drexel University, Philadelphia, USA).

The included articles indicate that a total of 266 organizations participated in the tACS study. As shown in Table 2, the institution with the highest number of publications in the field of tACS was the Carl von Ossietzky University of Oldenburg (50), followed by the University of Gottingen (44), and Harvard University (33). Germany, the USA, and the UK each accounted for 3 of the top 10 institutions. In terms of BC, the University of Gottingen (BC = 0.22) ranked first, followed by Harvard University (BC = 0.15) and the Max Planck Society (BC = 0.13) following suit (Figure 4).

**TABLE 2:** Top 10 institutions by publications.

Rank	Institution	Count	BC	Country
1	Carl von Ossietzky University of Oldenburg	50	0.10	Germany
2	University of Gottingen	44	0.22	Germany
3	Harvard University	33	0.13	USA
4	Max Planck Society	26	0.15	Germany
5	University of London	24	0.04	England
6	Sapienza University Rome	24	0.02	Italy
7	University of Oxford	23	0.10	England
8	University of North Carolina	23	0.02	USA
9	University of North Carolina Chapel Hill	23	0.02	USA
10	University College London	22	0.03	England

BC, betweenness centrality.

### Journal analysis

A total of 677 articles were published in 173 journals. According to Bradford’s law, 7 journals were considered core journals in the field (Table 3), with Brain Stimulation having the highest number of publications (58). This was followed by Frontiers in Human Neuroscience (48) and Scientific Reports (33). Brain Stimulation had the highest impact factor, followed by Neuroimage (IF = 4.7) and Journal of Neuroscience (IF = 4.4). In the quartile classification, 4 of the 7 journals were in the top 25% of the impact factor distribution across regions (Q1), 2 were in Q2, and 1 was in Q3.

**TABLE 3:** Top 7 journals and co-cited journals related to tACS.

Citing journals					Cited journals				
**Rank**	**Journal**	**Publication**	**IF**	**Quartile**	**Rank**	**Journal**	**Co-cited times**	**IF**	**Quartile**
1	Brain Stimulation	58	7.6	Q1	1	Brain Stimulation	617	7.6	Q1
2	Frontiers in Human Neuroscience	48	2.4	Q2	2	Journal of Neuroscience	590	4.4	Q1
3	Scientific Reports	33	3.8	Q1	3	Neuroimage	567	4.7	Q1
4	Neuroimage	28	4.7	Q1	4	Frontiers in Human Neuroscience	533	2.4	Q2
5	Frontiers in Neuroscience	27	3.2	Q2	5	Current Biology	520	8.1	Q1
6	Journal of Neuroscience	18	4.4	Q1	6	Clinical Neurophysiology	520	3.7	Q1
7	Brain Sciences	16	2.7	Q3	7	PLoS One	472	2.9	Q1

**FIGURE 5:**
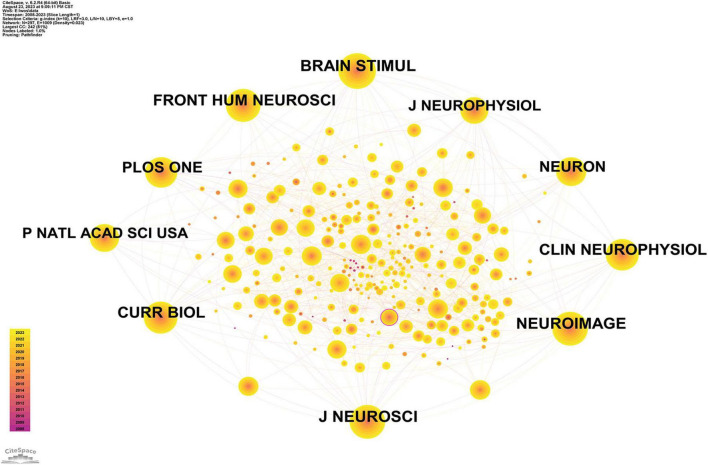
Journal co-cited network analysis. Time slicing: January 1999 to December 2023, Slice length: 1 year, Node types: Cited Journal, g-index (*K* = 12), Pruning: Pathfinder, *N* = 291, *E* = 1,596 (Software: CiteSpace 6.2.R4, Drexel University, Philadelphia, USA).

The highest co-citation frequency was also Brain Stimulation (617), followed by Brain Stimulation and Neuroimage (567). Of the 7 cited journals, 6 belonged to Q1 and the other 1 belonged to Q2 (Table 3). However, the BC values of the top 7 journals were all below 0.1 (Figure 5). The results suggest that Brain Stimulation and Neuroimage are in the field of tACS. The number of publications and citation frequency is high and the co-occurrence is large.

### References

As shown in Supplementary Table 1, 6 of the top 10 articles are from JCR Q1. The highest ranked academic literature has an impact factor of 14.7 points. Of the top 10 literature references, 4 studies focused on brain changes after tACS treatment. There are also two highly cited papers by Antal A in the top 10, namely “Safety, ethical, legal, regulatory and application guidelines for low-intensity transcranial electrical stimulation” and “Transcranial Alternating Current Stimulation (tACS).”

### Authors

#### Author publication volume analysis

Among the included articles, a total of 298 authors published tACS-related studies. As shown in Supplementary Table 2, Herrmann, CS had the highest number of publications (40), followed by Antal, A (22), Paulus, W (21). It is worth noting that the top 3 authors were all from Germany. In terms of centrality, the top 4 authors were all greater than 0.1.

#### Co-citation analysis

**FIGURE 6:**
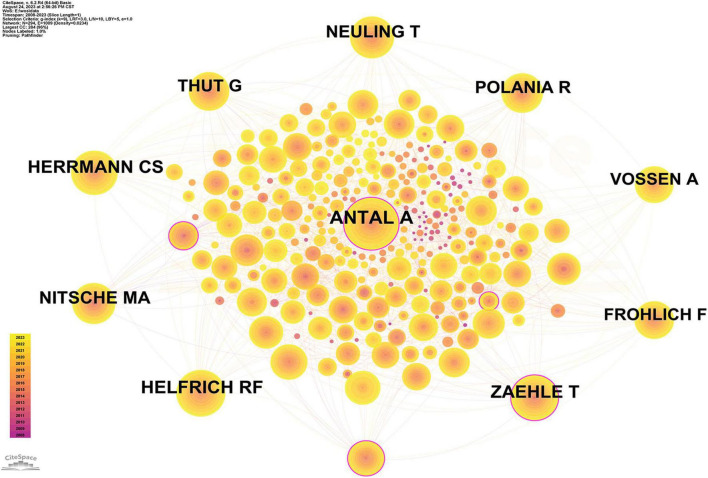
Author co-cited analysis (Software: CiteSpace 6.2.R4, Drexel University, Philadelphia, USA).

A co-citation relationship between authors occurs when two (or more) authors are simultaneously cited in one or more subsequent papers. Analyzing the co-citation network of authors gives a clear picture of the core authors and their contribution to a field. Notably, Herrmann, CS and Antal, A, Frohlich, F, Nitsche MA were among the top 10 in both the co-citation analysis and the collaborative network analysis (Figure 6). Antal A (0.16) and Zaehle T (0.14) led the way in centrality BC (Supplementary Table 3).

### Keywords

#### Keyword co-occurrence analysis

**FIGURE 7:**
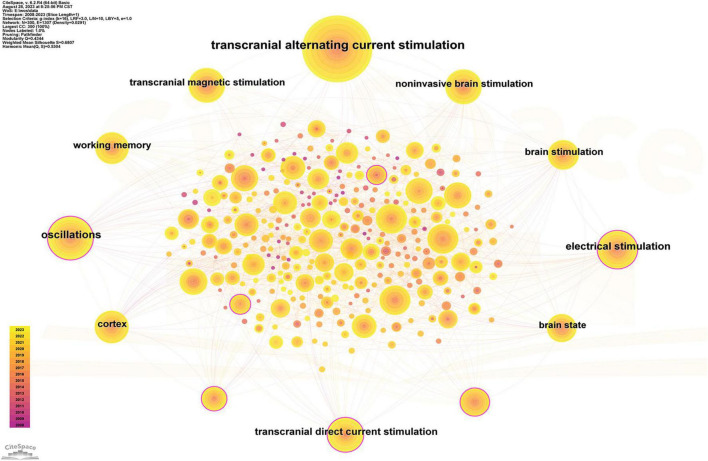
Keyword co-occurrence knowledge map. Keyword co-occurrence analysis. Time slicing: January 1999 to December 2023, Slice length: 1 year, Node types: Keyword, g-index (*K* = 12), Pruning: Pathfinder, *N* = 283, *E* = 1,239 (Software: CiteSpace 6.2.R4 Drexel University, Philadelphia, USA).

After merging the keywords, a total of 294 keywords appeared in the included articles. As shown in Supplementary Table 4, among the keywords with the highest co-occurrence frequency, in addition to “transcranial alternating current stimulation” (367), “oscillations” (170), “electrical stimulation” (137), “transcranial direct current stimulation” (112), and “cortex” (107) appeared more than 100 times. Key nodes (BC ≥ 0.1) for keyword co-occurrence analysis included “oscillations,” “electrical stimulation,” and “transcranial direct current stimulation” (Figure 7).

#### Keyword clustering analysis

**FIGURE 8:**
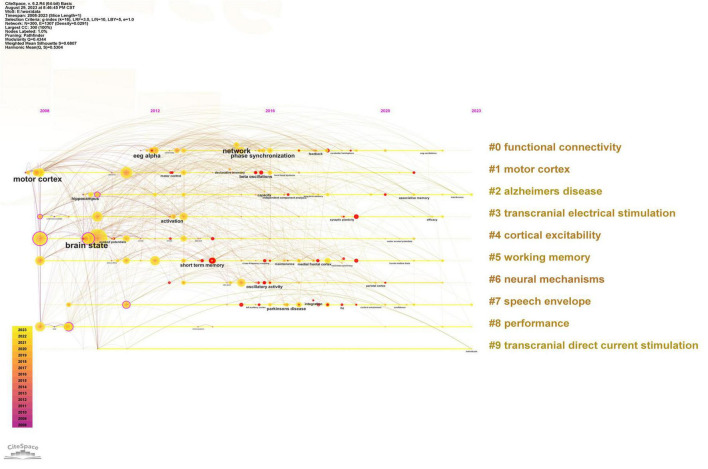
Keyword clustering and visual time-zone mapping (Software: CiteSpace 6.2.R4, Drexel University, Philadelphia, USA).

The keywords were analyzed and clustered using the Log Likelihood Ratio (LLR) algorithm, and the resulting clusters are shown in Figure 8. The cluster labels are shown on the right side, while the main time of occurrence for each keyword is shown on the horizontal axis. In addition, the connection between the keywords illustrates their overlapping relationship. The field of tACS research was divided into 10 clusters (Supplementary Table 5), with the top 3 being “functional connectivity” (33), “motor cortex” (27), and “Alzheimer’s disease” (25).

#### Keyword burst analysis

**FIGURE 9:**
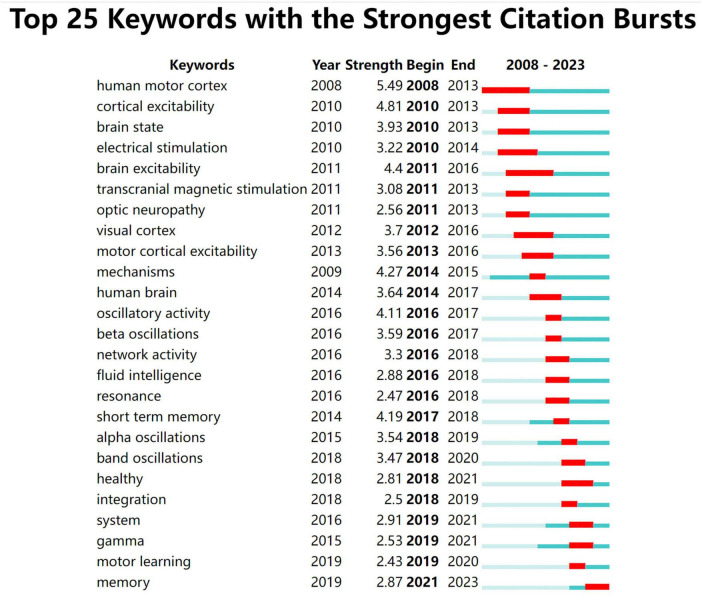
Top 25 keyword bursts (Software: CiteSpace 6.2.R4, Drexel University, Philadelphia, USA).

Keyword Burst Analysis shows that there is a tendency for keywords to change rapidly or increase significantly within a particular duration. The duration of the keyword after its sudden appearance is indicated by the red area, while the blue area indicates the period when the keyword was less frequently used. The saliency analysis obtained 25 keywords (Figure 9). The figure shows that “human motor cortex” (5.49), “cortical excitability” (4.81), “brain excitability” (4.4), and “mechanisms” (4.27) had “high saliency.” However, the bursts of relevant keywords lasted shorter, with the longest lasting for 6 years. As of 4 August 2023, the keyword that was still exploding was “memory.”

## Discussion

This paper focuses on the current state and hotspots of tACS research to date. 677 relevant publications matching the research topics were collected from the WoSCC database and analyzed using the CiteSpace software.

The analysis of related publications from different perspectives yielded different results. First, in terms of the quantity of publications, tACS research has appeared since 2008. From 2012 onward, the number of publications fluctuated, but showed an overall upward trend. This indicated that research in the field of tACS is promising. Secondly, the geographical distribution of publications showed that the developed countries of Germany, the USA, Italy and the UK were leading in terms of the number of articles published. We believe that this may be related to the strong economies and research environments of the developed countries. Among the top 10 institutions producing the most publications, Germany, the USA, and the UK each had three institutions. These findings illustrate the significant contribution of the three countries in this field. More attention could be paid to studies from the three countries by researchers working in this area.

The analysis of journals and co-cited journals can help the researcher to choose the right journal for submission. A co-citation relationship occurs when two journals are simultaneously cited in the same publication. Journal co-citation analysis can determine the impact of journals in the field of study (Liu et al., 2019). The number of published articles and co-citation results were analyzed. Brain Stimulation and Neuroimage are in the field of tACS. Researchers can pay more attention to the journals with high citation frequency to understand the latest information of tACS research in a timely manner.

The number of publications represents the authors’ contribution to the field of research, and the number of citations by co-citing authors reflects the authors’ influence. Antal A, Herrmann CS, Nitsche Ma, and Frohlich F, were ranked among the top 10 authors by number of publications and citations. The rankings show their significant contribution to the field of tDCS. In particular, the article “Entrainment of brain oscillations by transcranial alternating current stimulation” by Herrmann CS occupied the first position in co-cited references. This study employed EEG to record the synchronization of neuronal entrainment during tACS stimulation, while successfully distinguishing stimulation artifacts from ongoing and event-related cortical activity. Researchers can refer to the published works of these prolific authors to understand the hotspots in the field of tACS. It is also possible to grasp the cutting edge of the tACS field by studying the research directions of these prolific authors.

Keywords are a highly condensed representation of research topics (Ai et al., 2022). Keywords co-occurrence, keywords cluster and keywords burst analysis allow us to quickly and directly identify common themes and hotspots in the tACS field. In our analysis, “oscillations,” “electrical stimulation,” and “cortex” have received a great deal of attention from researchers. Meanwhile, “functional connectivity,” “motor cortex,” and “Alzheimer’s disease” remained active topics of long-term interest. This observation indicates that “Disease,” “neural mechanisms of the brain,” and “Electric stimulation” are significant hotspots in the tACS field, and we would explain this result from these three aspects.

As shown in the above results, tACS is often used for some diseases, including stroke, AD, and neuropsychiatric diseases. It is mainly because tACS can modulate neural activity and behavior in humans, which has great potential in the cognitive research and brain disorder therapies (Wischnewski et al., 2023). Currently, the clinical application of tACS in stroke rehabilitation focuses on motor dysfunction rehabilitation, post-stroke depression rehabilitation, and cognitive impairment rehabilitation (Schuhmann et al., 2022; Yang et al., 2023). Multiple studies have found that γ-tACS can effectively restore γ oscillations and improve AD symptoms, such as cognitive decline, working memory impairment, and behavioral changes (Benussi et al., 2021, 2022; Bréchet et al., 2021; Dhaynaut et al., 2022; Zhou et al., 2022; Nissim et al., 2023). TACS has also been used with partial efficacy in depression, schizophrenia, dementia, and other psychiatric disorders (Alexander et al., 2019; Sreeraj et al., 2019; Kraft and Hampstead, 2023). However, the exact mechanism of tACS has not been fully elucidated in these diseases. Future research should consider the following aspects: (1) With regard to the different dysfunctions and pathological features of the above diseases, it is necessary to screen the best therapeutic parameters and stimulation sites in order to obtain the most optimized neuromodulation effect. (2) There is a need to further validate the efficacy of tACS in these diseases through multicenter, large-sample, double-blind, randomized controlled studies. (3) It is essential to explore the action mechanism of tACS from different dimensions. It is believed that with the in-depth research of neuromodulation technology and further understanding of brain function, tACS is expected to become a precise and effective rehabilitation therapy.

Current research is also focused on understanding the neural mechanisms underlying tACS. In Liu et al. (2018) distinguished five mechanisms to explain the effects of tACS on neuronal and network activity: resonance, rhythmic resonance, temporal biasing of neuronal spikes, entrainment of network patterns, and imposed patterns. However, due to the different targets and diseases of interest, there are different explanations for the neural mechanism of tACS in different studies. In patients with major depression disorder, previous studies have shown that tACS increases levels of β-endorphin and serotonin in the cerebrospinal fluid, hypothalamus, and cortex, resulting in antidepressant effects (Zaghi et al., 2010). A randomized controlled trial used tACS with 10 Hz, 40 Hz, and sham stimulation interventions in the frontal lobes of three groups of major depressive disorder (MDD) patients. The 10 Hz tACS group showed a significant reduction in alpha power in the left frontal and central regions (Alexander et al., 2019). There have also been studies on the issue of γ oscillations and θ synchronizations in MDD patients (Wang et al., 2020a; McAleer et al., 2023b), but further mechanisms need to be explored. In PD patients, the improvement in motor cortex plasticity was due to enhanced γ oscillations by tACS during intermittent theta-burst stimulation (iTBS), which could restore impaired M1 plasticity and defective GABA-A-ergic inhibition (Guerra et al., 2023). Animal models of PD have found that when tACS acts on M1, it can activate endogenous glial cell line-derived neurotrophic factor in striatal parvalbumin-positive interneurons (GDNF) production and its survival signals exert protective effects on dopaminergic neurons in the substantia nigra (Lee et al., 2022). At present, studies have found that specific tACS programs can deliver electrical current to deep brain tissues, providing a reference for the regulation or treatment of neuropsychiatric disorders related to the hippocampus, insula, and amygdala (Shan et al., 2023).

Most of the current studies on the efficacy of tACS have focused on the electrical brain oscillations induced by tACS treatment (Schreglmann et al., 2021). Different brain diseases may induce different frequencies of abnormal brain oscillations, and tACS may work by modulating these abnormal oscillations (Antal and Paulus, 2013; Huang et al., 2021). Conventional tACS typically uses single-target stimulation to modulate a single region of brain activity. However, many clinical studies have confirmed the existence of functional connectivity and synchronization of neural activity between different brain regions (Chen et al., 2022). This provides a theoretical basis for multi-site stimulation in tACS. A study of dual-site tACS in healthy subjects found that after simultaneous stimulation of the right inferior frontal gyrus and center (midline) of the pre-supplementary motor area, the online and offline dual- site beta tACS can be beneficial in improving inhibitory control via distinct underlying mechanisms (Fujiyama et al., 2023). On the other hand, it has been reported that in the gamma band (30–100 Hz), communication between cortical regions is observed in the form of synchronous coupling, also known as the communication through coherence (CTC) hypothesis (Fries, 2005). According to the CTC hypothesis, multi-site tACS has been introduced to modulate synchronization between multiple brain regions. However, the reported results are confounding; tACS-induced synchronization of two cortical regions improved shape perception 8 and working memory while auditory motor mapping and right ear advantage were unaffected (Polanía et al., 2012; Meier et al., 2019; Preisig et al., 2019). In addition to tACS stimulation alone, researchers have attempted to combine tACS with other techniques to achieve better results. Using intermittent theta-burst stimulation (iTBS) in combination with gamma (γ) tACS stimulation and sham tACS stimulation during the on and off phases after oral levodopa administration in PD patients, it was found that regardless of dopaminergic status, enhancement of cortical γ oscillatory activity by tACS during iTBS improved M1 plasticity in PD patients and enhanced GABA-A-ergic transmission in PD patients regardless of dopaminergic status (Guerra et al., 2023). MRI, EEG, and functional near-infrared were used in the study to explore in depth the mechanism of action of tACS (Derks et al., 2024; Lu et al., 2024; Seo et al., 2024). In the future, multiple techniques may be combined to more comprehensively explore the effects of tACS on brain function and structure.

### Strengths and limitations

This study is the first to use bibliometrics to summarize and analyze the development trends and research hotspots of tACS. However, this study has several limitations. First, due to the limitations of CiteSpace software and databases, only the WoSCC database was analyzed in this study. Although most of the literature is included in the WoSCC database, the literature included in our study may not be exhaustive. Second, this study was limited the language to English, which may have overlooked literature in other language types.

## Conclusion

First, the treatment of tACS in diseases such as stroke, AD and schizophrenia remains a major clinical concern. Second, the neural mechanism of tACS to the brain still needs to be further explored. Third, as a promising NIBS, the combination of tACS with other techniques, such as therapeutic techniques and brain imaging assessment techniques, can further explore the exact role of tACS mechanisms and therapeutic effects.

## Data availability statement

The original contributions presented in this study are included in this article/Supplementary material, further inquiries can be directed to the corresponding authors.

## Ethics statement

Written informed consent was obtained from the individual(s) for the publication of any potentially identifiable images or data included in this article.

## Author contributions

C-FW: Data curation, Investigation, Software, Visualization, Writing – original draft. CS: Data curation, Investigation, Software, Visualization, Writing – original draft. Z-DW: Data curation, Investigation, Supervision, Validation, Visualization, Writing – review & editing. YG: Formal analysis, Supervision, Validation, Writing – review & editing. L-HZ: Investigation, Project administration, Validation, Writing – review & editing. W-JQ: Formal analysis, Project administration, Supervision, Validation, Writing – review & editing. TT: Conceptualization, Methodology, Project administration, Supervision, Validation, Visualization, Writing – review & editing.
