# Supplementary material for: A bibliometric analysis of transcranial alternating current stimulation
Source: Front Neurosci. 2024 Aug 2;18:1409492. doi: 10.3389/fnins.2024.1409492 (PMC11328521; doi:10.3389/fnins.2024.1409492)
Supplement: Supplementary file 1 [file Table_1.DOCX]

Supplementary Material

**Supplementary Table 1.** Top 10 references ranked by co-cited frequency.

| Rank | Title | Co-cited  frequency | BC | Journal | IF (2023) | JCR | Year |
| --- | --- | --- | --- | --- | --- | --- | --- |
| 1 | Entrainment of Brain Oscillations by Transcranial Alternating Current Stimulation | 150 | 0.1 | Current Biology | 8.1 | Q1 | 2014 |
| 2 | Alpha Power Increase After Transcranial Alternating Current Stimulation at Alpha Frequency (alpha-tACS) Reflects Plastic Changes Rather Than Entrainment | 111 | 0.07 | Brain Stimulation | 7.6 | Q1 | 2014 |
| 3 | Transcranial alternating current stimulation: a review of the underlying mechanisms and modulation of cognitive processes | 100 | 0.02 | Frontiers In Human Neuroscience | 2.4 | Q2 | 2013 |
| 4 | Orchestrating neuronal networks: sustained after-effects of transcranial alternating current stimulation depend upon brain states | 88 | 0.12 | Frontiers In Human Neuroscience | 2.4 | Q2 | 2013 |
| 5 | Low intensity transcranial electric stimulation: Safety, ethical, legal regulatory and application guidelines | 88 | 0.04 | Clinical Neurophysiology | 3.7 | Q1 | 2017 |
| 6 | Sustained Aftereffect of alpha-tACS Lasts Up to 70 min after Stimulation | 77 | 0.22 | Frontiers In Human Neuroscience | 2.4 | Q2 | 2016 |
| 7 | Direct effects of transcranial electric stimulation on brain circuits in rats and humans | 75 | 0.04 | Nature Communications | 14.7 | Q1 | 2018 |
| 8 | The Importance of Timing in Segregated Theta Phase-Coupling for Cognitive Performance | 74 | 0.04 | Current Biology | 8.1 | Q1 | 2012 |
| 9 | tACS motor system effects can be caused by transcutaneous stimulation of peripheral nerves | 66 | 0.08 | Nature Communications | 14.7 | Q1 | 2019 |
| 10 | Transcranial alternating current stimulation (tACS) | 66 | 0.02 | Frontiers In Human Neuroscience | 2.4 | Q2 | 2013 |

BC, betweenness centrality; IF, impact factor; JCR, Journal Citation Reports.

**Supplementary Table 2.** Top 9 authors by publications.

| Rank | Author | Count | Country | BC | Year |
| --- | --- | --- | --- | --- | --- |
| 1 | Herrmann, CS | 40 | Germany | 0.15 | 2010 |
| 2 | Antal, A | 22 | Germany | 0.22 | 2008 |
| 3 | Paulus, W | 21 | Germany | 0.14 | 2008 |
| 4 | Frohlich, F | 16 | USA | 0.11 | 2018 |
| 5 | Nitsche, MA | 14 | Germany | 0.09 | 2012 |
| 6 | Guerra, A | 12 | Italy | 0.01 | 2018 |
| 6 | Berardelli, Alfredo | 12 | Italy | 0.01 | 2018 |
| 8 | Engel, AK | 10 | Germany | 0.01 | 2014 |
| 9 | Santarnecchi, E | 9 | USA | 0.05 | 2013 |
| 9 | Rossi, S | 9 | Italy | 0 | 2013 |
| 9 | Sack, Alexander T | 9 | Netherlands | 0.01 | 2015 |
| 9 | Kasten, Florian H | 9 | Germany | 0.07 | 2018 |

BC, betweenness centrality.

**Supplementary Table 3.** Top 10 authors ranked by co-cited frequency.

| Rank | Author | co-cited frequency | BC |
| --- | --- | --- | --- |
| 1 | Antal A | 391 | 0.16 |
| 2 | Helfrich Rf | 310 | 0.08 |
| 3 | Zaehle T | 286 | 0.14 |
| 4 | Herrmann Cs | 284 | 0.07 |
| 5 | Nitsche MA | 247 | 0.06 |
| 6 | Neuling T | 238 | 0.09 |
| 7 | Polania R | 229 | 0.07 |
| 8 | Thut G | 214 | 0.08 |
| 9 | Frohlich F | 203 | 0.05 |
| 10 | Vossen A | 197 | 0.04 |

BC, betweenness centrality.

**Supplementary Table 4.** Top 10 keywords ranked by number of occurrences.

| Rank | Keywords | Number of  Occurrences | BC | Year of  Occurrence |
| --- | --- | --- | --- | --- |
| 1 | transcranial alternating current stimulation | 367 | 0.06 | 2010 |
| 2 | oscillations | 170 | 0.14 | 2008 |
| 3 | electrical stimulation | 137 | 0.1 | 2010 |
| 4 | transcranial direct current stimulation | 112 | 0.11 | 2010 |
| 5 | cortex | 107 | 0.09 | 2008 |
| 6 | transcranial magnetic stimulation | 100 | 0.02 | 2011 |
| 7 | noninvasive brain stimulation | 99 | 0.06 | 2010 |
| 8 | brain stimulation | 92 | 0.08 | 2008 |
| 9 | working memory | 91 | 0.05 | 2012 |
| 10 | brain state | 83 | 0.09 | 2010 |

BC, betweenness centrality.

**Supplementary Table 5.** List of keyword clusters.

| Rank | Size | Silhouette | Cluster | Keywords (Partial) |
| --- | --- | --- | --- | --- |
| #0 | 33 | 0.626 | functional connectivity | alpha oscillations; phase synchronization; entrainment; deep brain stimulation |
| #1 | 27 | 0.714 | motor cortex | excitability; working memory; transcranial magnetic stimulation; declarative memory |
| #2 | 25 | 0.55 | alzheimers disease | dementia; associative memory; dorsolateral prefrontal cortex (dlpfc); mild cognitive impairment |
| #3 | 18 | 0.705 | transcranial electrical stimulation | transcranial electric stimulation; oscillations; non-invasive brain stimulation; safety |
| #4 | 27 | 0.77 | cortical excitability | transcranial alternating current stimulation; alternating current stimulation; transcranial magnetic stimulation; oscillations |
| #5 | 31 | 0.684 | working memory | theta frequency; brain stimulation; cognitive control; theta oscillations |
| #6 | 16 | 0.596 | neural mechanisms | enhancement; speech; alpha power; short term memory |
| #7 | 22 | 0.665 | speech envelope | brain oscillations; speech-shaped-noise; speech comprehension; normal hearing |
| #8 | 9 | 0.893 | performance | purdue pegboard test; supplementary motor area; somatosensory; objects |
| #9 | 4 | 0.93 | transcranial direct current stimulation | theta synchrony; internalizing psychopathologies; emotion regulation; sham stimulation |
